# Parathyroid Hormone Induces Bone Cell Motility and Loss of Mature Osteocyte Phenotype through L-Calcium Channel Dependent and Independent Mechanisms

**DOI:** 10.1371/journal.pone.0125731

**Published:** 2015-05-05

**Authors:** Matthew Prideaux, Sarah L. Dallas, Ning Zhao, Erica D. Johnsrud, Patricia A. Veno, Dayong Guo, Yuji Mishina, Stephen E. Harris, Lynda F. Bonewald

**Affiliations:** 1 Department of Oral and Craniofacial Sciences, School of Dentistry, University of Missouri-Kansas City, Kansas City, Missouri, United States of America; 2 Department of Biologic and Materials Sciences, School of Dentistry, University of Michigan, Ann Arbor, Michigan, United States of America; 3 Peridontics and Cellular and Structural Biology, University of Texas Health Science Center, San Antonio, Texas, United States of America; Université de Lyon - Université Jean Monnet, FRANCE

## Abstract

Parathyroid Hormone (PTH) can exert both anabolic and catabolic effects on the skeleton, potentially through expression of the PTH type1 receptor (PTH1R), which is highly expressed in osteocytes. To determine the cellular and molecular mechanisms responsible, we examined the effects of PTH on osteoblast to osteocyte differentiation using primary osteocytes and the IDG-SW3 murine cell line, which differentiate from osteoblast to osteocyte-like cells in vitro and express GFP under control of the dentin matrix 1 (*Dmp1*) promoter. PTH treatment resulted in an increase in some osteoblast and early osteocyte markers and a decrease in mature osteocyte marker expression. The gene expression profile of PTH-treated Day 28 IDG-SW3 cells was similar to PTH treated primary osteocytes. PTH treatment induced striking changes in the morphology of the *Dmp1*-GFP positive cells in IDG-SW3 cultures and primary cells from *Dmp1*-GFP transgenic mice. The cells changed from a more dendritic to an elongated morphology and showed increased cell motility. E11/gp38 has been shown to be important for cell migration, however, deletion of the *E11/gp38/podoplanin* gene had no effect on PTH-induced motility. The effects of PTH on motility were reproduced using cAMP, but not with protein kinase A (PKA), exchange proteins activated by cAMP (Epac), protein kinase C (PKC) or phosphatidylinositol-4,5-bisphosphonate 3-kinase (Pi3K) agonists nor were they blocked by their antagonists. However, the effects of PTH were mediated through calcium signaling, specifically through L-type channels normally expressed in osteoblasts but decreased in osteocytes. PTH was shown to increase expression of this channel, but decrease the T-type channel that is normally more highly expressed in osteocytes. Inhibition of L-type calcium channel activity attenuated the effects of PTH on cell morphology and motility but did not prevent the downregulation of mature osteocyte marker expression. Taken together, these results show that PTH induces loss of the mature osteocyte phenotype and promotes the motility of these cells. These two effects are mediated through different mechanisms. The loss of phenotype effect is independent and the cell motility effect is dependent on calcium signaling.

## Introduction

Osteocytes are the most abundant and long lived cells within the bone and are known to play important roles in regulating bone formation, resorption and homeostasis. They represent the terminal differentiation stage of the osteoblast lineage, where an osteoblast has become entrapped within the mineralized matrix. Although the location of osteocytes deep within the mineralized bone matrix has hindered investigation into their biology, several important functions of osteocytes have now become apparent (reviewed in [1]). Recent studies have indicated the importance of osteocytes in maintaining bone mass. They are important regulators of osteoclast formation and activity [2–5] and may be the primary source of receptor activator of nuclear factor kappa-B ligand within the adult skeleton [3,4]. Osteocytes also play an important role in controlling osteoblast differentiation via the expression of wnt signaling inhibitors such as sclerostin and dikkopf-related protein 1 [6–8]. Osteocytes are sensory cells and are very responsive to changes in their extracellular environment, such as mechanical strain (see [9,10] for review) and biochemical and hormonal signals (reviewed in [1,11]). One of the most important and well known of these signals is parathyroid hormone (PTH), which is secreted by the parathyroid gland and is known to have both anabolic and catabolic effects on the skeleton [12].

It has long been suggested that the osteocyte is a target cell for PTH. Changes in cytoskeletal ultrastructure and increased microfilament and microtubule formation were observed in osteocytes treated with PTH *in vivo* [13,14]. The PTH receptor, PTH1R, is present on osteocytes *in vivo* [15,16] in addition to osteoblasts, but is absent from osteoclasts, suggesting that PTH regulation of bone resorption is mediated by cells other than the osteoclast itself. PTH1R is also present on primary osteocytes *in vitro* and primary osteocytes were found to be more responsive to PTH compared to osteoblasts [17]. PTH downregulates expression of the wnt antagonist sclerostin [18,19]. Sclerostin is a potent inhibitor of osteoblastic bone formation as deletion of sclerostin in mouse models results in increased bone mass [20]. The use of a monoclonal antibody targeting sclerostin has proved successful at increasing bone formation in animal models and clinical trials [21–23]. A murine model in which the PTH1R was constitutively activated in osteocytes under control of the dentin matrix 1 (*Dmp1*) promoter resulted in increased bone formation and resorption with a net increase in bone mass [24].

One of the difficulties in studying the effects of PTH on osteocytes is their relative inaccessibility. Cell lines have been made, such as MLO-Y4 and MLO-A5 cells. While they are useful for mechanistic studies, these cells have their limitations. MLO-Y4 cells are more representative of an early osteocyte and do not reside within a mineralized matrix or express mature osteocyte markers [25,26]. MLO-A5 cells do synthesize a mineralized matrix but only have very low basal levels of *Sost* expression [26–28]. A novel, conditionally immortalized cell line, IDG-SW3, has recently been developed in our laboratory, which recapitulates differentiation from an osteoblast to a mature osteocyte over a twenty eight day culture period. These cells initially have an osteoblastic phenotype, but when cultured under mineralizing conditions express early osteocyte markers such as E11/podoplanin, followed by *Dmp1* and finally by mature markers such as sclerostin and fibroblast growth factor 23 (*Fgf23*) by 21–28 days of culture [29]. These cells also express green fluorescent protein (GFP) under control of the 8kb *Dmp1* promoter while they are mineralizing and respond to hormonal signals such as PTH by decreasing *Sost* expression and to 1,25(OH)_2_D_3_ by increasing *Fgf23* expression, in a similar fashion to osteocytes *in vivo* [29,30].

To further understand the mechanisms underlying the effects of PTH in bone, the IDG-SW3 cell line was used in the present study to investigate the effects of PTH on osteoblasts/osteocytes at different stages of differentiation. Mature IDG-SW3 cells (representing the late osteocyte phenotype) and primary osteocytes were particularly sensitive to PTH treatment and lost their mature osteocyte phenotype. Cells positive for *Dmp1*-GFP representing early osteocytes responded to PTH with a dramatic change in morphology and an increase in motility. This change was mediated by cAMP generation and was regulated by Ca^2+^ signaling through the L-Type calcium channel. These studies therefore describe a new function for this channel in modulating the effects of PTH on bone cells.

## Materials and Methods

### Cell culture

IDG-SW3 cells were cultured as described previously [29]. These cells are immortalized with a temperature sensitive T-antigen that is induced by interferon gamma (IFN-γ), therefore they proliferate under permissive conditions (33°C in α-MEM with 10% FBS, 100 U/ml penicillin, 50μg/ml streptomycin) (all Hyclone, South Logan, UT) and 50 U/ml IFN-γ (Life Technologies, Carlsbad, CA) on type I collagen coated plates. For experiments, cells were plated at a density of 4x10^4^ cells/cm^2^ in collagen coated 6 or 12-well plates (Corning, Tewksbury, MA). Once the cultures reached confluence, the media was replaced with osteogenic media (α-MEM with 10% FBS, 100 U/ml penicillin, 50 μg/ml streptomycin, 50μg/ml ascorbic acid and 4mM β-glycerophosphate) (both Sigma-Aldrich, St. Louis, MO) in the absence of IFN-γ and cells were cultured at 37°C. Media was changed every three days.

To examine the effects of PTH on IDG-SW3 cells, the media was replaced with fresh osteogenic media and the cultures were treated with a range of concentrations of bovine PTH (1–34) (1–100nM) (Sigma) or vehicle (PBS) for either 24 or 48 hours. Cultures were also treated with Forskolin (1–50 μM) (Sigma) and 8-bromo-cAMP (1–100μM) (Sigma), a stable isoform of cAMP. To investigate the pathways mediating the effects of PTH, IDG-SW3 cultures were treated with PKI 14–22 (Tocris Bioscience, Bristol, UK), 6-Bnz-cAMP (Biolog, Bremen, Germany), 8-CPT-2Me-cAMP (Tocris), phorbol 12-myristate 13-acetate (PMA) (Sigma), Go6983 (Tocris), PKC-zeta pseudosubstrate (Tocris), LY294002 (Sigma), Wortmannin (Tocris), Nifedipin (Tocris) and Diltiazem Hydrochloride (Tocris), or vehicle controls, at the indicated concentrations.

### Culture of *ex-vivo* osteocyte enriched bone fragments and whole bone

Long bones (tibia, femur and humerus) were aseptically dissected from 4 individual male 4 month old C57/BL/6 mice, euthanized by carbon monoxide treatment followed by cervical dislocation. The bones were cleaned of muscle, the epiphyses were removed and the marrow flushed out with a syringe and 27 gauge needle. The bones were dissected into 2mm sized pieces and subjected to three 25 minute sequential digestions in 2mg/ml Type IA collagenase (from Clostridium histolyticum, Sigma) in α-MEM and 5mM EDTA/0.1% BSA (Sigma) in PBS as described in [31], to remove cells from the bone surface. After digestion, the bone pieces from each mouse were further dissected into smaller fragments (approx. 1mm^2^), divided between 2 wells of a 12 well plate and cultured overnight in αMEM supplemented with 10% FBS, 100 U/ml penicillin and 50μg/ml streptomycin. The following day the bone fragments from each mouse were treated with either 50nM PTH or PBS (as a vehicle control) and cultured for a further 24 hours before harvesting for RNA extraction for qPCR analyses. All animal experiments were performed with the approval of the Institutional Animal Care and Use Committee at the University of Missouri, Kansas City and conformed to relevant federal guidelines.

To obtain a gene expression profile of the effects of PTH on osteocytes within their mineralized micro-environment, long bones from 2–3 month old mice were used. The mice were sacrificed by cervical dislocation, the epiphyses were removed just below the growth plate, the marrow flushed out as above, and then the bones were digested with 0.2% Type I Collagenase (Sigma) and 0.05% trypsin (Sigma) in αMEM plus antibiotics, for 30 min with rotation at 150rpm at 37 degrees. The marrow region from each bone, tibia plus femur, was again flushed with media until all the marrow was removed. The bones were then digested for 20 min each, 3 times with the collagenase-trypsin mixture as above. By histology these bone cylinders or bone “tubes” are free of any osteoblasts on the periosteal or endosteal surface and contain no growth plate material. The tubes contain predominantly osteocytes in their “natural” bone matrix environment *ex vivo*. The osteocyte bone tubes were cultured in αMEM plus 10% FBS plus antibiotics overnight. The media was changed the next day with or without 250nM PTH to ensure penetration within the bone matrix. The bone tubes were removed after 24 hours, rinsed with PBS, and frozen until RNA extraction for array analyses.

### RNA Extraction of *Ex Vivo* Bone “tubes” for Global Gene Expression Profiling

The frozen tibia and femurs, from 4–5 mice per experiment, were placed in liquid nitrogen and pulverized with 3–5 strokes. To the frozen bone powder, approximately 10 bones per preparation, 5ml of Trizol (Life Technologies) was added and allowed to come to room temperature (approx. 1hr). The mixture was placed in a glass-teflon homogenizer and given 10 strokes. The RNA was then extracted following the manufacturer’s instructions and biotinylated cRNA prepared with the Ambion cRNA kit (Life Technologies). The cRNAs were then hybridized with the Mouse Illumina v6 array with 45,281 probes (Illumina, San Diego, CA), washed and scanned with the Illumina scanning system. The data files were then analyzed with the Genome Studio RNA expression module, using quantile normalization and a differential expression score of >5 or <-5 (p<0.05) that takes into consideration multiple testing issues (see Genome Studio Manual).

### RNA Seq Analysis of PTH-treated Mature Day 28 IDG-SW3 Cells

IDG-SW3 cells were differentiated for 28 days in one well of a 6 well plate in osteogenic media and then treated for 24 hours with either 50nM PTH or PBS in triplicate. Total RNA was isolated using Trizol according to the manufacturer’s instructions. The transcriptome profile of the Day 28 IDG-SW3 cells treated with and without PTH was determined by RNA sequencing using the HiSeq 2000 sequencing platform (Illumina). Sample amplification, labeling, hybridization and scanning were carried out by the microarray core of the University of Missouri-Columbia. Libraries were constructed following the manufacturer’s protocol with reagents supplied in Illumina’s TruSeq RNA sample preparation kit v2. Briefly, the poly-A containing mRNA is purified from total RNA, RNA is fragmented, double-stranded cDNA is generated from fragmented RNA, and the index containing adapters are ligated to the ends. Total RNA (2ug) was first incubated in a thermal cycler for 5 minutes at 65°C in a total volume of 50ul in a 96-well PCR plate. The plate was removed and incubated an additional 5 minutes at room temperature allowing RNA to bind to the poly-T oligo-attached magnetic beads. Beads were washed by placing the PCR plate on the magnetic stand at room temperature for 5 minutes and discarding supernatant. Bead Washing Buffer (200ul) was added and returned to the magnetic stand for 5 minutes. Supernatant was removed and discarded. The plate was removed from the magnetic stand and Elution Buffer (50ul) was added to each well. The plate was incubated at 80°C for 2 minutes and then placed at room temperature. RNA was rebound to beads with the addition of Bead Binding Buffer (50ul) and incubated for 5 minutes at room temperature. Beads were washed as previously described. First strand cDNA synthesis was performed by adding the Elute, Prime, Fragment Mix (19.5ul) to each well. The mixture was incubated for 8 minutes at 94°C. The plate was placed on the magnetic stand at room temperature for 5 minutes. From the plate, 17ul of the fragmented and primed RNA was transferred to a new PCR plate. First Strand Master Mix and Superscript II mix (8ul) was added to each well and gently mixed. Incubation was performed in a thermal cycler with the program: 25°C^(10:00)^+42°C^(50:00)^+70°C^(15:00)^. Second strand cDNA synthesis was performed by the addition of Second Strand Master Mix (25ul) to each well. Mixture was incubated at 16°C for 1 hour. Aline PCRClean beads (90ul) were added to each well containing 50ul of ds cDNA. The plate was incubated at room temperature for 15 minutes and placed on the magnetic stand for 5 minutes. The supernatant (135ul) was removed and discarded. Each well was washed by addition of 200ul of 80% EtOH, incubation at room temperature for 30 seconds, and removal of supernatant. Wash steps were repeated once and plate was allowed to dry on magnetic stand for 15 minutes. Resuspension Buffer (52.5ul) was added to each well. The plate was returned to the magnetic stand at room temperature for 5 minutes and 50ul of supernatant was transferred to a new PCR plate. Fragment overhang ends were converted to blunt ends by the addition of the End Repair Mix (40ul) to each well and incubation at 30°C for 30 minutes. Aline PCRClean beads (160ul) were added to each well which contained 100ul of End Repair Mix. Plate was incubated at room temperature for 15 minutes. Supernatant (127.5ul) was removed and discarded. Each well was washed with 80% EtOH as previously described. The dried pellet was resuspended in Resuspension Buffer (20ul) and 15ul was transferred to a new PCR plate. The 3’ ends of the fragments were adenylated with the addition of A-Tailing Mix (12.5ul) to each well and then incubated for 30 minutes at 37°C. DNA Ligase Mix (2.5ul) and a single RNA Adapter Mix (2.5ul) were added to each well and then incubated for 10 minutes at 37°C. The ligation reaction was stopped with the addition of Stop Ligase Mix (5ul). Aline PCRClean beads (42ul) were added to each well. The plate was incubated at room temperature for 15 minutes. Supernatant (79.5ul) was removed and discarded. Each well was washed with 80% EtOH as previously described. The dried pellet was resuspended in Resuspension Buffer (52.5ul) and 50ul was transferred to a new PCR plate. Aline PCRClean beads (50ul) were added to each well. The plate was incubated at room temperature for 15 minutes. Supernatant (95ul) was removed and discarded. Each well was washed with 80% EtOH as previously described. The dried pellet was resuspended in Resuspension Buffer (22.5ul) and 20ul was transferred to a new PCR plate. DNA fragments were enriched by adding PCR Primer Cocktail (5ul) and PCR Master Mix (25ul) to each well. PCR amplification was performed as follows: 98°C^(0:30)^+[98°C^(0:10)^+60°C^(0:30)^+72°C^(0:30)^] x 15 cycles +72°C^(5:00)^. The amplified cDNA construct were purified by addition of Aline PCRClean beads (50ul) to each well. The plate was incubated at room temperature for 15 minutes. Supernatant (95ul) was removed and discarded. Each well was washed with 80% EtOH as previously described. The dried pellet was resuspended in Resuspension Buffer (32.5ul), incubated at room temperature for 2 minutes, and then placed on the magnetic stand for 5 minutes. Supernatant (30ul) was transferred to low binding microcentrifuge tube for storage. The final construct of each purified library was evaluated using the BioAnalyzer 2100 automated electrophoresis system, quantified with the Qubit flourometer using the quant-iT HS dsDNA reagent kit (Invitrogen), and diluted according to Illumina’s standard sequencing protocol for sequencing on the HiSeq 2000. The data sets can be found under: http://www.ncbi.nlm.nih.gov/geo/query/acc.cgi?acc=gse61351.

### Comparison of IDG-SW3 PTH responsive genes with long bone osteocyte PTH responsive genes

Gene Set Enrichment analysis was used to compare the PTH responsive genes between the two datasets. A non-redundant file of 33,277 probes with n = 3 for control and n = 3 for PTH treatment was used to construct a. gct file dataset, and the 1794 PTH responsive genes in the IDG-SW3 cell model was used to construct a. gmx file geneset. The phenotype file,. cls, was PTH vs Control. These files were then analyzed using the program, GSEA, at the Broad Institute. A. gmx file with only the positive PTH responsive genes in the IDG-SW3 cell model was prepared and compared with the dataset containing all the non-redundant data with the 33,277 probes from the bone tube osteocyte analysis. This was again analyzed with the GSEA program.

### Real-time PCR

Total RNA was isolated from IDG-SW3 cultures (in triplicate wells of a 12-well plate) and cultured bone fragments (from 4 individual mice) in Trizol according to the manufacturer’s instructions. RNA was treated with DNAse I (Life Technologies) to remove genomic DNA contaminants and 1μg was reverse transcribed into cDNA using the high capacity cDNA kit (Life Technologies) according to the manufacturer’s instructions. Real-time PCR was performed using 25ng of template cDNA with Taqman gene arrays and 2x Master mix in duplicate on a Step One Plus cycler (all Life Technologies). The data was normalized to the housekeeping gene *Actb*. Relative expression was determined using the 2^-ΔΔCt^ method [32].

### Western blotting

IDG-SW3 cells were cultured in 12 well plates in osteogenic media as described previously. 48 hours before harvesting, the media on the cells was replaced with fresh osteogenic media containing 50nM PTH or PBS. After 48 hours, the media was removed from the cultures and the cell monolayers were rinsed twice in PBS and lysed in ice-cold RIPA buffer (50mM Tris, 150mM NaCl, 0.5% sodium deoxycholate, 1% NP-40) containing Complete Mini protease inhibitors (Roche, Indianapolis, IN). All steps were performed on ice to prevent protein degradation. 10μg of total protein from each sample was loaded onto a 10% Bis-tris gel (Biorad, Hercules, CA) and separated by SDS PAGE as described previously [33]. The protein was electroblotted to a PVDF membrane (GE Healthcare, Pittsburg, PA) for 45 minutes at 360mA. 8.1.1. antibody [34] was used to detect E11 expression (1:2500 dilution of hybridoma culture supernatant) and polyclonal anti-sclerostin antibody AF1589 (R&D Systems) was used to detect sclerostin (1:500 dilution) along with the appropriate horseradish peroxidase (HRP)-conjugated secondary antibodies. Blots were stripped using Restore stripping buffer (Thermo Fisher Scientific, Rockford, IL) and re-probed using a monoclonal, HRP-conjugated β-actin antibody (1:25,000 dilution) (Sigma) as a loading control. Immunoreactive bands were detected using the SuperSignal West Dura Chemilumienescence kit (Thermo Fisher Scientific). Densitometry was performed using a Fujifilm LAS 4000 gel documentation system in conjunction with the Multi-gauge software (Fujifilm, Tokyo, Japan).

### Quantification of GFP expression

Protein lysates were harvested from IDG-SW3 cultures as described above. 50μl of lysate was measured in a Victor II fluorescent plate reader (PerkinElmer, Waltham, MA) and the background fluorescence, from a blank of lysis buffer, was subtracted. For analysis, the relative fluorescent units (RFU) were normalized to total protein concentration, measured using BCA assay according to manufacturer’s instructions (Thermo Fisher Scientific).

### Microscopy

To investigate GFP expression and cell morphology, images were taken of the cultures using a Nikon TE300 microscope under epifluorescence illumination (10x objective/100ms exposure) with a Photometrics Coolsap EZ cooled CCD camera interfaced with the PM Capture Pro software (Photometrics, Tucson, AZ).

For confocal microscopy, the SW3 cultures were grown on glass coverslips and mounted onto a glass slide using 50% glycerol:50% PBS + 1mM MgCl_2_. The samples were then imaged a using a Leica TCS Sp5 II laser scanning confocal microscope interfaced with the LAS AF software (Leica Microsystems, Wetzlar, Germany). Images were captured using a 40x objective and a Z stack was created from 45 planes with a distance of 0.5μm between planes.

### Timelapse Imaging of Cell Motility

Cells were plated at a density of 4x10^4^/cm^2^ on collagen coated 12 well plates and differentiated for 28 days. The cultures were then supplemented with the indicated treatments and timelapse imaging was performed as described previously [35] for 48–72 hours using a Nikon TE 2000E microscope under epifluorescence illumination. The microscope system is fully automated, with precision motorized x, y and z stage. The “Metamorph” software (Molecular Devices, LLC, Sunnyvale, CA) was used to control the microscope hardware and multidimensional imaging parameters. During imaging, the temperature was held constant at 37°C and a humidified 5% CO_2_ atmosphere was maintained as described previously [35]. For simultaneous imaging of bone mineral together with *Dmp1*-GFP positive cells, cultures were supplemented with 0.5ug/ml alizarin red as a vital stain for calcium. Images were acquired for each time point under epifluorescent illumination (10x objective) using a Photometrics Coolsnap HQ cooled CCD camera with 12-bit grey scale resolution. Fields of 895 x 668μm were imaged at a spatial resolution of 696 x 520 pixels (2x2 binned mode) every 30 minutes for up to 72 hours from 5–7 optical planes. Timelapse image stacks were processed in Metamorph using the “best focus” algorithm and exported as 8 bit image stack (.stk) files. Contrast adjustment and red-green merging was performed using Image J software (downloadable from: http://rsb.info.nih.gov/ij/). Single color or merged image stacks were also processed using the “stackreg” plugin in Image J, which registers/aligns the stack of image slices to correct for rigid body motion [36]. The Dmp1-GFP timelapse movie stacks were normalized using the Image J normalization function to allow continued visualization of the cell shape changes/motility in PTH-treated samples with time in culture even though the Dmp1-GFP expression was reduced. Image stacks were then assembled into movies using Image J software and converted to mp4 files using Windows Movie Maker (Microsoft, Redmond, WA). To quantify the motility of *Dmp1*-GFP positive cells, individual cell trajectories were plotted frame by frame using the “MTrackJ” plugin in Image J (Erik Meijering, Biomedical Imaging Group, Erasmus MC—University Medical Center Rotterdam). The software then calculates distance moved and velocity of the cell. For analysis, 30–40 cells were selected at random in three separate fields and the mean velocity was determined for the cells in each movie field, to give three independent data points (n = 3).

### Generation of E11-hypomorphic mice

Mice in which part of exon 1 and intron 1 of the *E11* gene were floxed were generated as described below. A floxed E11/gp38 construct was created from three source plasmids. Plasmid 1 contains a 12 kb SacI to SalI mouse genomic DNA of *E11*/*gp38* promoter, exon 1 and partial intron 1 carried by pBluescript SK vector (Stratagene, La Jolla, CA, USA). Plasmid 2 is a loxp site inserted into pBluescript SK at SmaI and BamHI. Plasmid 3 includes a Flipped RNA polymerase II promoter driving neomycin resistant gene followed by a loxP site (FRT-polIIneor- FRT-loxP) in the vector of pUC9 (Sigma-Aldrich, St. Louis, MO, USA). FRT-polIIneor-FRT was used as a retrievable neomycin selection marker of ES cells. From plasmid 1, a 3.6 kb fragment containing the promoter and partial exon 1 non-coding region of *E11*/*gp38* gene was released by SalI and EcoICRI, and inserted into plasmid 2 at the 5’ side of the loxp site by SalI and EcoRV. The resulting plasmid was further linearized by SacI, and ligated to a 3.5 kb fragment containing 3’ partial exon 1 and intron 1 of the *E11*/*gp38* gene released by SacI from plasmid 1. Then, the resulting plasmid was linearized by BstEII in intron 1, and ligated to the 3.4 kb FRT-polIIneor- FRT-loxp cassette released by SpeI and ClaI from plasmid 3. Both of the fragments were filled-in as blunt ends before ligation. This final plasmid was linearized by SalI before electroporation into ES cells. Each step was confirmed by restriction mapping (S1 Fig). The final construct was also validated by sequencing at every joint of ligation. The primer used to confirm the 5’ loxP site was 5’-ATAAATGCCGACTGTGC-3’. The primer used to confirm the 5’ FRT was 5’-GGGCTAAGTCTCCTGTAACA-3’. The primer used to confirm the polII was 5’-TACAGAATGGCCCTAACAAC-3’. The primers used to confirm the 3’ loxP and FRT sites were 5’-GTGGAATGGGTTGGTAGAG-3’ and 5’- ATTCTCAGGCTCCATTCG-3’. All the enzymes were from New England Biolabs, Ipswich, MA, USA. Theoretical DNA sequence analysis and primer design were conducted with Gene Tool (BioTools, Edmonton, Alberta, Canada) and MacVector (MacVector, Cary, NC, USA). DNA Sequencing was performed by Eurofins MWG Operon, Huntsville, AL, USA.

The linearized targeting vector was electroporated into 107 AB2.2 ES cells (Lexicon Genetics, The Woodlands, Texas, USA) and 1.6 x 10^7^ clone A3 of UG347 ES cells established from 129SvEv blastocysts. Three hundred G418-resistant ES cell clones from each cell line, a total of 600 clones, were initially screened by Southern Blotting and 33 correctly targeted ES cell clones were identified. Twenty-four were confirmed to possess the loxP site in intron 1. The targeted ES clones were injected into blastocysts from C57BL/6 albino mice. The resulting chimeras were bred to C57BL/6 females and F1 agouti offspring were genotyped by Southern analyses. Five targeted clones were used for injection and two of them underwent germline transmission (S2 Fig).

These mice were discovered to express only 10% of the normal levels of *E11* mRNA and undetectable levels of E11 protein (unpublished data) due to disruption of exon 1 resulting in a hypomorphic *E11* allele. This resulted in an E11-deficient phenotype without the need for Cre-recombination. To produce E11-deficient mice in which osteocyte differentiation could be tracked using a fluorescent reporter gene, *E11* floxed mice were bred with mice expressing GFP under control of the 8kb *Dmp1* promoter (provided by Dr. Ivo Kalajzic and Dr. David Rowe, University of Connecticut) [37] to obtain mice that expressed *Dmp1*-GFP on a background that was homozygous for the *E11* floxed (hypomorphic) allele.

### Culture of E11-Hypomorphic Dmp1 GFP Primary Calvarial Cells

5 day old *E11*-hypomorphic *Dmp1* GFP and *E11*-expressing *Dmp1* GFP mice were sacrificed by cervical dislocation and primary osteoblasts were isolated from the calvaria using collagenase/trypsin digestions as described in [38]. The cells were plated at a density of 2x10^4^ cm^2^ in α-MEM supplemented with 10% FBS. Once they reached confluence, the cells were cultured in osteogenic media (α-MEM, 10% FBS, 50μg/ml ascorbic acid and 4mM βGP) for 21 days. This induces differentiation of foci of *Dmp1*-GFP positive cells that form mineralized bone nodules. The cultures were then treated with 50nM PTH or vehicle control (PBS) and time lapse imaging was performed as described above.

### Statistical Analysis

A one way ANOVA was performed for experiments in which there were more than two treatment groups to compare, such as time course experiments with and without PTH treatment. This was followed by a Bonferroni post hoc test to compare differences between groups. In situations where there were only two groups for comparison, a Student’s t-test was used. All analyses were performed using Prism 5 (GraphPad, La Jolla, CA). A value of p<0.05 was considered significant.

## Results

### PTH Induces Loss of Mature Osteocyte Phenotype

**Fig 1:**
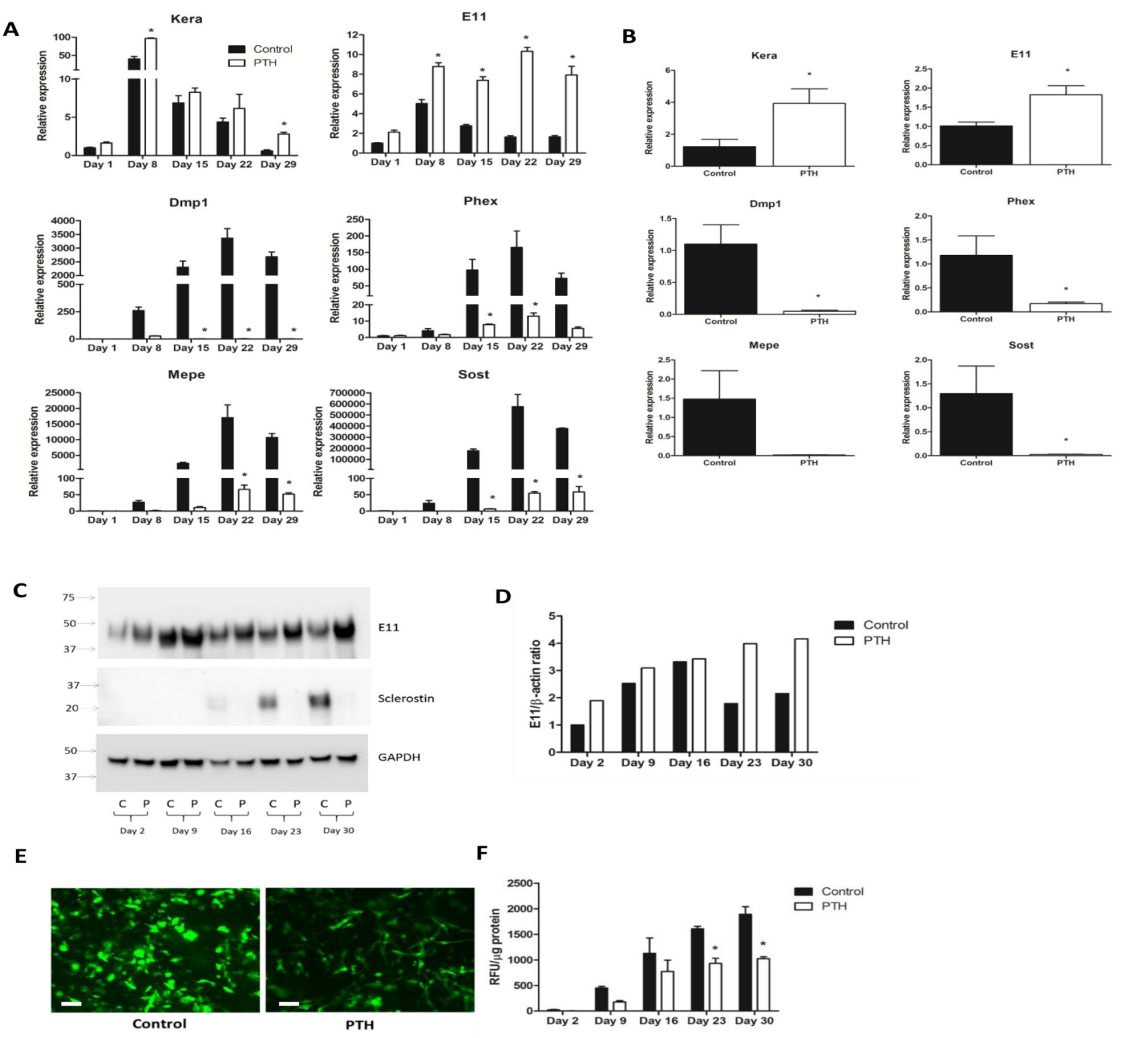
The effect of PTH on osteoblast and osteocyte marker gene and protein expression in IDG-SW3 cells and primary osteocytes. **(A)** Real-time PCR analysis of IDG-SW3 cells cultured over a 29 day time course and treated with 50nM PTH, or PBS control, for 24 hours at days 0, 7, 14, 21 and 28. Expression was normalized to *Actb* and is relative to day 1 control samples. *Kera* and *E11* were upregulated by PTH treatment, whereas *Dmp1*, *Phex*, *Mepe* and *Sost* expression was decreased, particularly at the later time points (n = 3±SD, *p<0.05). **(B)** Real-time PCR analysis of osteocyte-enriched bone fragments cultured in the presence of 50nM PTH, or PBS, for 24 hours. Expression was normalized to *Actb* and is relative to control samples. *Kera* and *E11* were upregulated by PTH treatment. *Dmp1*, *Phex*, *Mepe* and *Sost* expression was decreased in the primary osteocytes (n = 4±SD, *p<0.05). **(C)** IDG-SW3 cells were cultured over a 30 day time course and treated with 50nM PTH, or PBS, for 48 hours at days 0, 7, 14, 21 and 28. E11 and sclerostin expression was assessed by western blotting. **(D)** Quantitation of E11 protein expression with and without PTH treatment, relative to day 2 control samples. **(E)** GFP expression in mature (day 28) IDG-SW3 cells treated with 50nM PTH for 48 hours. Scale bar = 20μm. **(F)** Quantification of GFP in IDG-SW3 cells cultured over a 30 day time course and treated with 50nM PTH for 48 hours at days 0, 7, 14, 21 and 28 (n = 4±SD, *p<0.05).

To determine the effects of PTH on osteoblast and osteocyte gene expression, IDG-SW3 cells were treated at day 0, 7, 14, 21 and 28 with 50nM PTH for 24 hours and gene expression was examined by RT-PCR. Under control conditions, the osteoblast marker *Kera*, which encodes for the gene keratocan, was highly expressed in the immature cells at day 8 of culture and was downregulated in the mature, more differentiated IDG-SW3 cells. The early osteocyte marker *E11/gp38/pdpn* was also highly expressed in the early stages of differentiation and its expression decreased in the mature cultures (Fig 1A). PTH treatment increased the expression of both markers, with a particularly potent effect on increasing *E11* expression even at the latest time points. The osteocyte markers *Dmp1*, matrix extracellular phosphoglycoprotein (*Mepe*), phosphate-regulating gene with homologies to endopeptidases on the X chromosome (*Phex*) and *Sost* were all expressed at low levels or absent at day 1 but their expression increased with differentiation in the control cultures as reported previously [29]. A single treatment with PTH for 24 hours significantly decreased the expression of these genes to very low levels. In the case of *Dmp1*, the expression was completely blocked by PTH treatment.

Primary bone osteocytes were used to validate observations using IDG-SW3 cells (Fig 1B). Similar effects were also observed. *Kera* and *E11* expression were increased by supplementation with PTH for 24 hours, whereas *Dmp1*, *Phex*, *Mepe* and *Sost* were all downregulated by PTH compared to vehicle control.

Protein expression correlated with mRNA as 50nM PTH also induced a robust increase in E11 protein expression in mature IDG-SW3 cells after 48 hours treatment (Fig 1C and 1D). Sclerostin expression, which was first observed by day 16 of differentiation in the control cultures, was dramatically downregulated by PTH treatment so that it was undetectable by western blotting and thus was not quantifiable (Fig 1C). PTH also decreased the levels of GFP in the IDG-SW3 cultures (Fig 1E and 1F), consistent with the effects of PTH on *Dmp1* expression seen in Fig 1A and 1B.

### Comparison of IDG-SW3 with long bone osteocyte gene expression profile using GSEA

431 genes were found to be responsive to PTH in the *ex vivo* bone osteocytes cultured overnight. A cut off of 1.5 fold change either positive or negative was set. Many of the PTH negative response genes are the same as in the IDG-SW3 cell culture and represent genes considered to be osteoblast/osteocyte enriched, such as *Sost*, *Mef2c*, *Mepe*, *Phex*, *Dlx3*, *Ibsp*, *Osteocalcin/Bglap*, *Col1a1*, *Pth1r*. The raw data, and analyzed data sets (n = 3 for Control and n = 3 for PTH treated) can be found in the ncbi GEO database, with accession number GSE61146. Many of the positive PTH responsive genes are involved in bone resorption, such as *Tnfsf11*, *Oscar*, and *Nfac4* and dendrite formation, such as *E11/GP38* and *Sema4f*.

Next the PTH response dataset derived from the RNA-seq analysis of Day 28 PTH treated IDG-SW3 cells (1794) was compared with the PTH response in the ex vivo osteocyte bone tube cultures. A non-redundant set of 3 controls and 3 PTH treated RNA samples with 33,277 gene probes was prepared for Illumina Mouse W6 v2 arrays. Next the Gene Set Enrichment program at the Broad Institute (GSEA) was used to determine if there is a set of genes in this 33,277 that are enriched in the PTH or control datasets of the IDG-SW3 cell model. 1334 of the 1794 PTH treated IDG-SW3 genes could be mapped to the PTH treated bone tube data. As shown in the Enrichment profile, there is both a positive enrichment and a strong negative enrichment set in the bone tube data with the PTH negative responsive genes in the IDG-SW3 cell model. The positive enrichment did not quite reach significance. The negative dataset was highly significant with a NES of- 1.44, and contained a core 365 genes that are negatively regulated by PTH in both the cell model and the bone tubes. This negative enrichment set includes many of the classical osteocyte and osteoblast selective genes, such as *Sost*, *Mef2c*, *Osx* (*Sp7*), *Col1a1*, *Osteocalcin/Bglap*, *Mepe*, *Dmp1*, *Dlx3*, *Opg*. The entire 365 geneset is in S1 Table.

**Fig 2:**
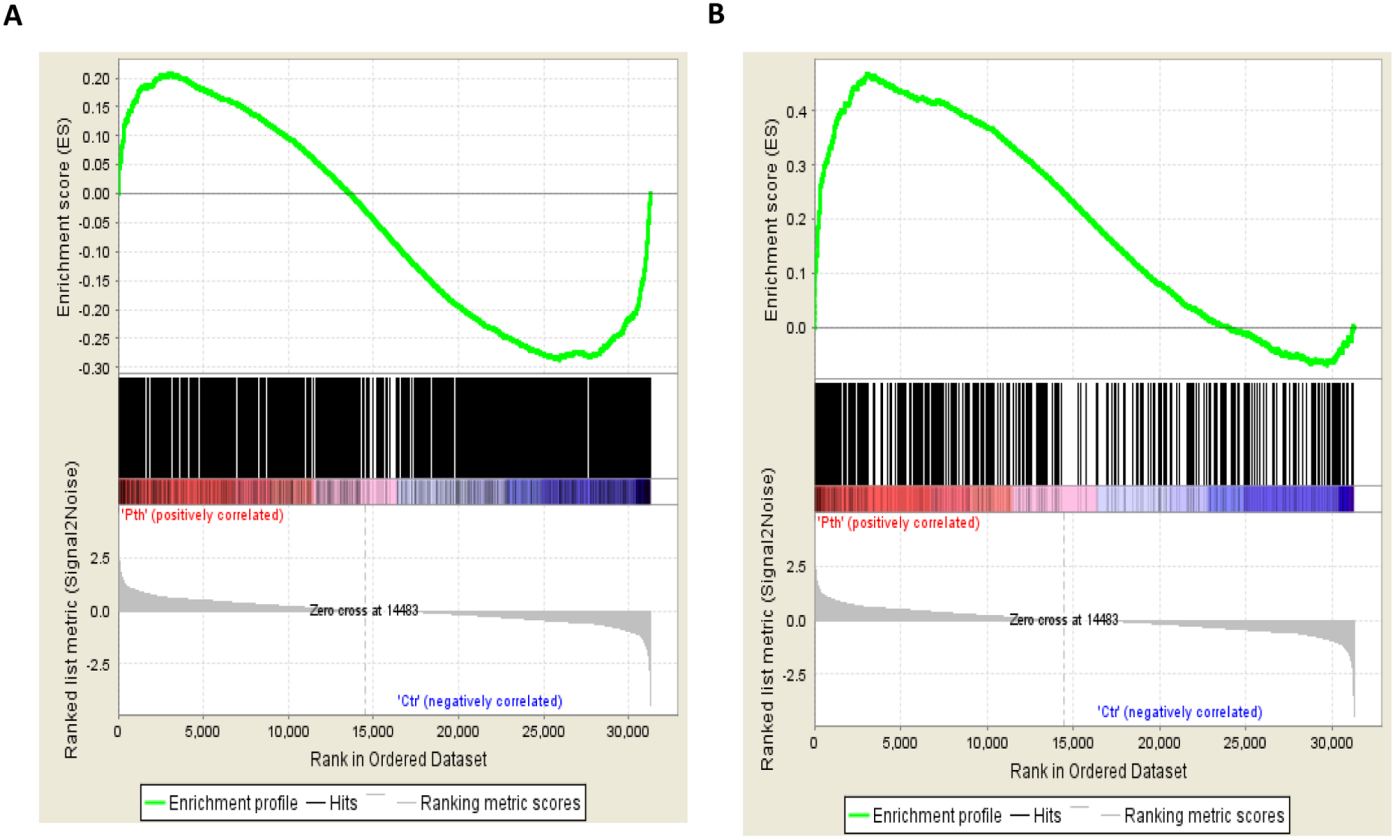
Gene set enrichment analysis of PTH Responses in the IDG-SW3 Osteocyte Enriched Cell model and *Ex Vivo* Cortical Bone Osteocyte Model. **(A)** 33,277 gene expression values were analyzed against the entire 1794 geneset of PTH responsive genes in the IDG-SW3 cell model. Highly significant enrichment (NES -1.44) was found in the genes in both models that were negatively responsive to PTH (365, see S1 Table). **(B)** The 574 genes that responded positively to PTH in the IDG-SW3 cell model, was analyzed against the 33,277 gene expression values in the *ex vivo* bone cortical osteocyte model, and a highly significant enrichment (NES = +1.98) was found in a set of genes that positively responded to PTH (131 geneset, see S2 Table).

Subsequently a file was made with only the positive PTH responsive genes in the IDG-SW3 cell model RNA-seq data. This 574 geneset was then compared with the complete 33,277 probe data in the bone tube experiments. A highly significant enrichment of 131 genes out of 440 mapped were found that were positively responsive to PTH in both the cell model and the bone tube model. These genes included pleiotrophin (*Ptn*), which is known to be involved in bone formation (see Fig 2 and list of 131 genes found with this analysis in S2 Table).

### PTH Induces Changes in Morphology and Increases Motility in IDG-SW3 Cells

**Fig 3:**
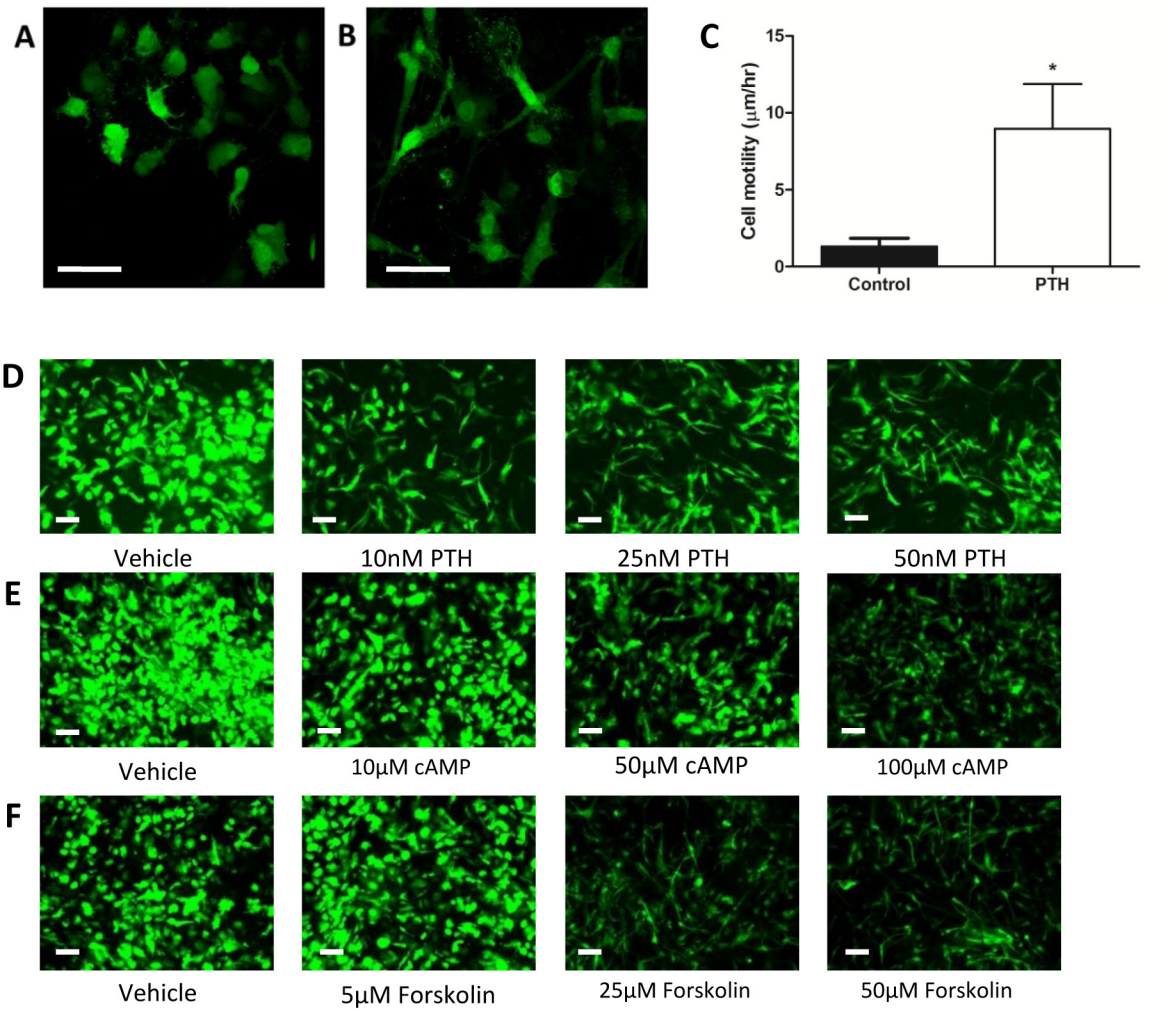
The effect of PTH on IDG-SW3 cell morphology and motility. Images of GFP positive day 30 IDG-SW3 cells treated with PBS (**A**) or 50nM PTH (**B**) for 48 hours and captured by confocal microscopy. Scale bar = 25μm. (**C**) Mean velocity of mature IDG-SW3 cell motility in response to 50nM PTH over a 63 hour time course. Representative time lapse images can be found in S1 Video (n = 3 observation fields±SD, with each field containing 30–40 cells, *p<0.05) Changes in IDG-SW3 cell morphology induced by PTH, cAMP and forskolin. Effect on mature IDG-SW3 cell morphology induced by 48 hours treatment with dose response of (**D**) PTH, (**E**) 8-bromo-cAMP and (**F**) forskolin. Scale bar = 20μm.

In addition to the changes in gene expression induced by PTH we also observed that the IDG-SW3 cells underwent a striking change in morphology. Mature (day 28) IDG-SW3 cultures were treated with 50nM PTH for 48 hours and examined by confocal microscopy. The GFP positive cells in the control cultures had a rounded morphology, with dendrites extending from the cell body (Fig 3A). Those in the PTH treated cultures, however, displayed an elongated, morphology (Fig 3B).

Changes in cell morphology are often associated with cell movement. To examine if PTH affected IDG-SW3 cell motility, mature (day 28) cultures were treated with 50nM PTH or vehicle control and their movement was observed using timelapse imaging. In the PTH treated cultures many of the GFP positive cells not only became elongated but became highly motile (S1 Video), whereas in the control cultures there were very few motile GFP positive cells. Cell motility was significantly increased with PTH treatment (Fig 3C). It is important to note, however, that not all of the PTH treated cells became motile and some, presumably those deeply encased within the mineralized matrix, remained immobile. The same effects of PTH on IDG-SW3 cells were observed with primary *Dmp1*-GFP cells (S2 Video).

### PTH Induced Changes in IDG-SW3 Morphology are Mimicked by cAMP and forskolin

Many of the effects of PTH are known to be mediated by the second messenger cAMP. To determine if increased cAMP generation is responsible for the changes in IDG-SW3 cell morphology we treated mature IDG-SW3 cultures with increasing concentrations of forskolin or 8-bromo-cAMP, a stable isoform of cAMP. Both forskolin and 8-bromo cAMP induced changes in IDG-SW3 cell morphology and *Dmp1*-GFP expression identical to those observed with PTH (Fig 3D, 3E and 3F).

### E11/gp38 does not play a role in PTH induced motility

**Fig 4:**
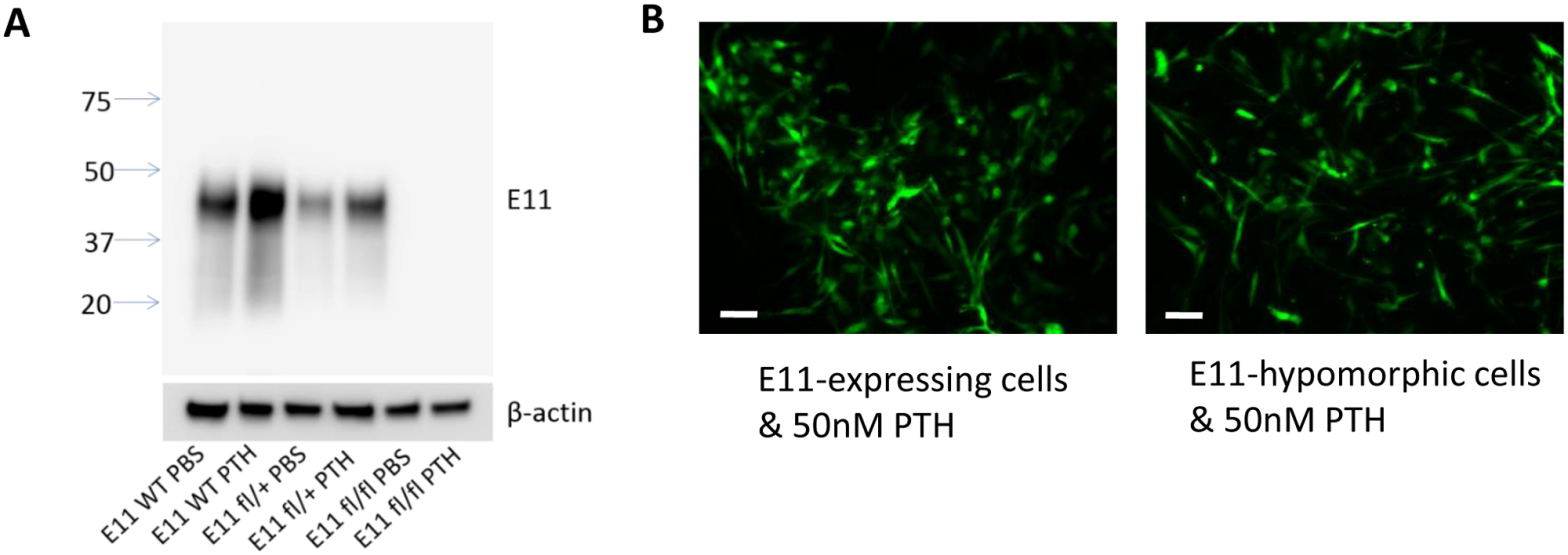
E11/gp38 is not involved in increased motility in response to PTH. (A). Primary osteocytes from E11-hypomorphic (fl/fl) mice do not express detectable E11 protein in the absence or presence of PTH, whereas E11 protein expression is induced in by PTH in E11-expressing cells. (B) PTH induces changes in cell morphology of both E11-expressing and E11-deficient primary osteocytes. Scale bar = 20μm.

To determine if the increase in E11 expression observed after PTH treatment could be responsible for the change in morphology and motility, calvarial cells were harvested from mice in which Exon 1 of *E11* gene had been floxed. Previously, we had shown that E11 was necessary for dendrite extension in response to fluid flow shear stress [39]. Unexpectedly, cells taken from these mice, in the absence of a Cre driver, had no detectable levels of E11 protein as analyzed by western blotting (Fig 4A), suggesting that insertion of the loxP sequences in the exon 1 region of the *E11* gene resulted in a hypomorphic allele with very low to negligible E11 expression, without the need for Cre recombination. Time lapse imaging was performed on primary calvarial cells from mice expressing the *Dmp1*-GFP transgene on the *E11*flx/flx (hypomorphic) background. The cells were differentiated for 21 days and then treated with PTH for 48 hours. Time lapse imaging showed that there was no difference in the cell elongation/motility response to PTH between the *E11*-hypomorphic *Dmp1* GFP cells compared to *E11*-expressing *Dmp1* GFP cells (Fig 4B, S2 Video). This suggests that E11 is not required for these effects of PTH on osteocyte-like cells. Analysis of the 131 geneset of the RNA-seq data from PTH treated IDG-SW3 cells with David Bioinformatics identified a subset of 11 genes known to be involved in cell motility that are upregulated by PTH treatment and could potentially be involved (Table 1).

**Table 1:** Fold change values of motility-associated genes upregulated by PTH treatment in IDG-SW3 cells and corresponding values in *ex vivo* cortical bone osteocytes as determined by David analysis.

Gene Symbol	Gene Name	Fold change IDG-SW3 cells	Fold change *ex-vivo* osteocytes
Gli3	GLI-Kruppel family member GLI3	2.84	1.37
Bdnf	brain derived neurotrophic factor	3.31	37.57
Egfr	epidermal growth factor receptor	3.46	1.01
Etv1	ets variant gene 1	4.65	1.08
Gdnf	glial cell line derived neurotrophic factor	7.44	1.53
Itga11	integrin alpha 11	3.68	1.17
Plat	plasminogen activator, tissue	5.77	1.58
Sema4f	sema domain, immunoglobulin domain (Ig), TM domain, and short cytoplasmic domain	5.17	1.64
Sema3c	sema domain, immunoglobulin domain (Ig), short basic domain, secreted, (semaphorin) 3C	6.65	16.69
Nck2	similar to SH2/SH3 adaptor protein; non-catalytic region of tyrosine kinase adaptor protein 2; predicted gene 6226	2.14	1.33
Tes	testis derived transcript	2.94	1.11

The full geneset can be found in S2 Table.

### PTH Induced Changes in IDG-SW3 Morphology are Independent of the PKA, Epac, PKC and Pi3K Pathways

**Fig 5:**
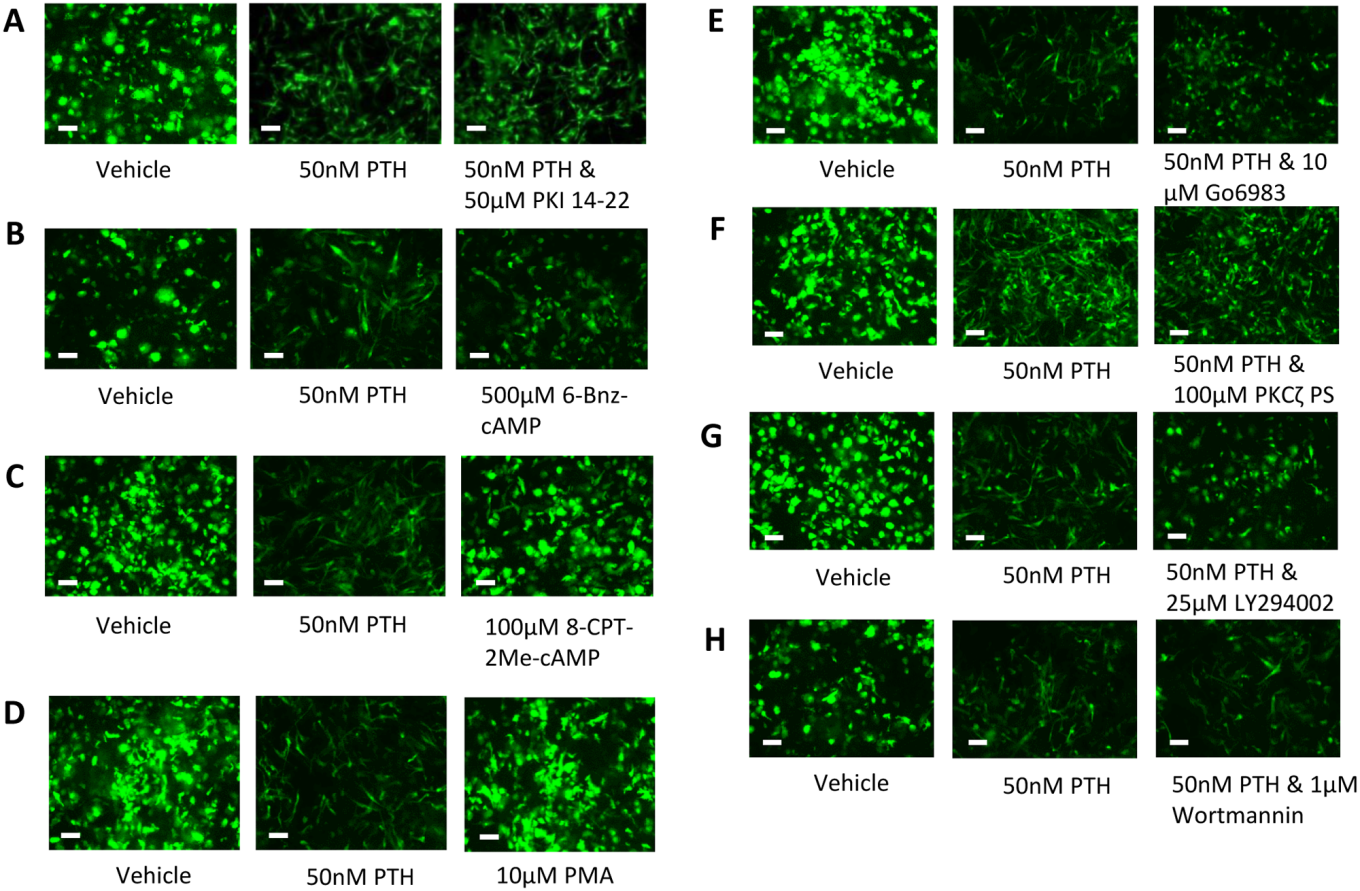
Lack of effect of inhibitors on PTH induced cell morphology. (A) The PKA inhibitor PKI 14–22 (50μM) does not block the effects of 50nM PTH on IDG-SW3 cell morphology. (B) Activation of the PKA pathway using 6-Bnz-cAMP does not reproduce the effect of PTH. (C) The Epac specific activator 8-CPT-2Me-cAMP (100μM) does not reproduce the effect of PTH on IDG-SW3 cell morphology. (D) The PKC activator PMA (100μM) does not affect IDG-SW3 cell morphology. (E) The broad spectrum PKC inhibitor Go6983 (10μM) blocked the effects of 50nM PTH on IDG-SW3 cell morphology. (F) The atypical PKC inhibitor PKC-zeta pseudosubstrate (PS) (100μM) failed to block the effect of 50nM PTH on IDG-SW3 cell morphology. (G) The Pi3K inhibitor LY294002 (25μM) blocked the effect of 50nM PTH on IDG-SW3 cell morphology, however the more potent and specific inhibitor Wortmannin (1μM) failed to do so (H). Scale bar = 20μm.

PTH and cAMP have been shown to affect many downstream signaling pathways associated with regulation of cell morphology and motility. One of the most common pathways activated by PTH/cAMP is the protein kinase A (PKA) pathway. However, we found that culturing IDG-SW3 cells with PTH in the presence of the PKA inhibitor PKI 14–22, failed to block the effects of PTH on cell morphology (Fig 5A). Furthermore, the PKA specific activator 6-Bnz-cAMP did not induce these morphological changes in IDG-SW3 cells even at concentrations of up to 500μM (Fig 5B), although it did reduce Dmp1-GFP expression in these cells. The exchange protein activated by the cAMP (Epac) family of small GTPases are also known to be activated by cAMP and regulate the cell cytoskeleton and motility [40]. However, the Epac specific activator 8-CPT-2Me-cAMP did not change IDG-SW3 cell morphology nor show any effect on *Dmp1*-GFP expression (Fig 5C), suggesting that Epacs are not involved in either the PTH effects on cell morphology or *Dmp1* expression. The protein kinase C (PKC) activator Phorbol 12-myristate 13-acetate (PMA) failed to induce morphological changes or downregulation of *Dmp1*-GFP in mature IDG-SW3 cells (Fig 5D). However the PKC inhibitor Go6983 was able to block the changes in morphology induced by PTH, but not the downregulation of *Dmp1*-GFP expression (Fig 5E). There are many members of the PKC family and the atypical PKCs are sensitive to Go6983 inhibition but are not activated by PMA [41]. To determine if the atypical PKC-zeta mediates the effects of PTH, we treated mature IDG-SW3 cells with PTH in the presence of the PKC-zeta pseudosubstrate. The pseudosubstrate failed to block the changes in cell morphology or the downregulation of *Dmp1*-GFP expression induced by PTH (Fig 5F).

Another pathway associated with cell morphology is the phosphoinositide 3-kinase (Pi3K) pathway. We found that the Pi3K inhibitor LY294002 was able to block the effects of PTH on IDG-SW3 cell morphology (Fig 5G). However, a more potent and selective Pi3K inhibitor, Wortmannin, was unable to replicate these effects (Fig 5H), suggesting that some effect of LY294002 other than its inhibition of Pi3K may be responsible for blocking the PTH-mediated change in morphology. Neither LY294002 nor Wortmannin were able to prevent the downregulation of *Dmp1*-GFP expression in response to PTH. All of the inhibitors and activators of the signaling pathways were tested using a range of doses, with the effects of the highest doses shown in Fig 5.

### Calcium Signaling Mediates the Effects of PTH on Cell Morphology

The ability of Go6983 and LY294002, inhibitors of PKC and Pi3K signaling respectively, to inhibit the changes in cell morphology were surprising as other PKC and Pi3K inhibitors were unable to replicate these effects. Interestingly, in addition to their effects on PKC and Pi3K, both Go6983 and LY294002 have been shown to target L-type calcium channel activity. Expression of the L-type calcium channel has been shown to be higher in osteoblasts than osteocytes and the T-type calcium channel to be higher in osteocytes than osteoblasts [42].

Upon examination of the RNA Seq gene array data of the IDG-SW3 cells, the L-type calcium channel subunit *Cacna1c* was found to be increased in expression by PTH treatment (1.5 fold) and the T-type calcium channel subunit *Cacna1h* was downregulated by PTH in mature IDG-SW3 cells by greater than two-fold.

**Fig 6:**
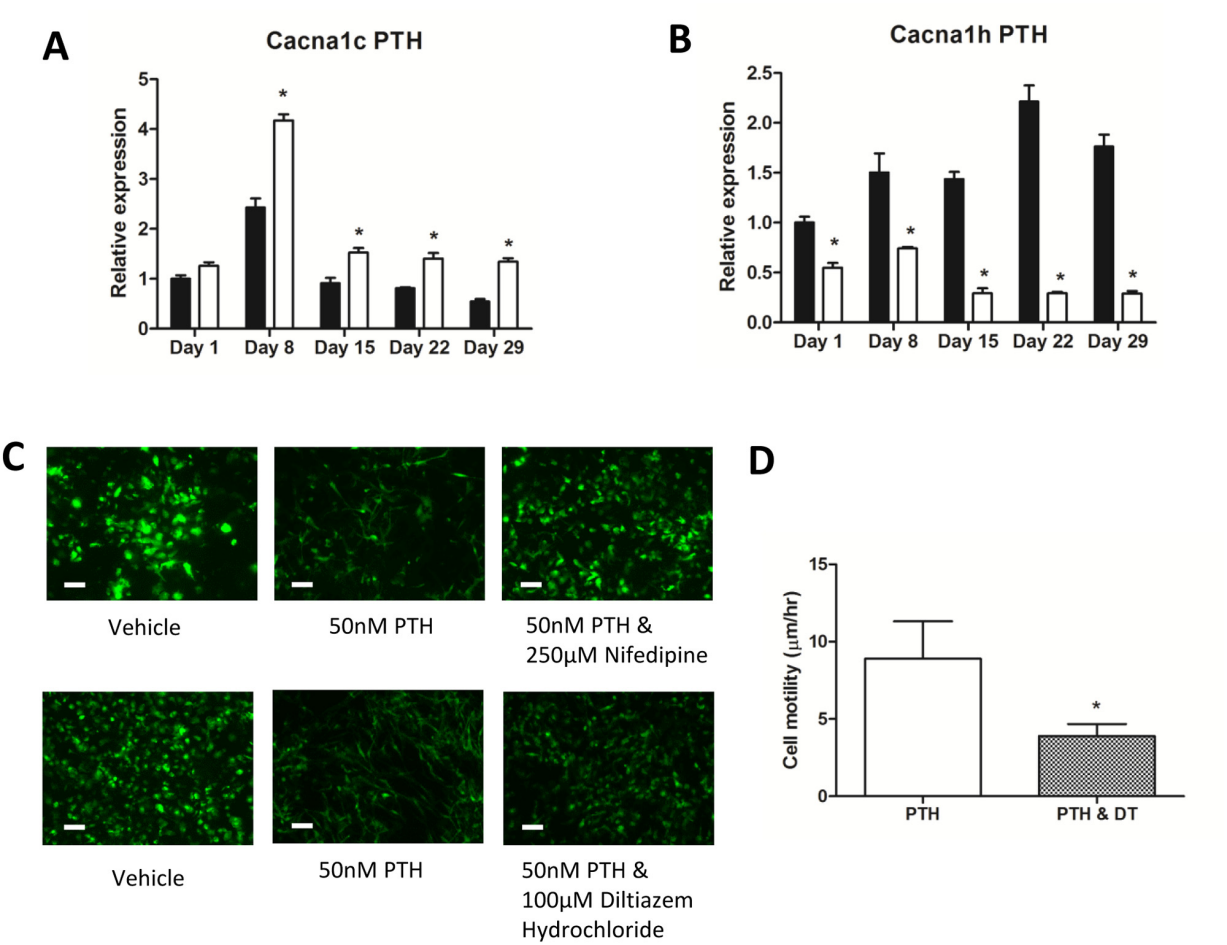
L-type calcium channels mediate the effects of PTH on IDG-SW3 cell morphology and motility. (A, B) Real-time PCR analysis showing expression of the L and T-type channel subunits in IDG-SW3 cells cultured over a 29 day time course and treated with 50nM PTH for 24 hours at days 0, 7, 14, 21 and 28. Expression was normalized to *Actb* and is relative to day 1 control samples. (C) The L-type calcium channel blockers Nifedipine (250μM) and Diltiazem (100μM) inhibit the effect of 50nM PTH on mature IDG-SW3 cell morphology. (D) Mean velocity of mature IDG-SW3 cells treated with 50nM PTH alone or 50nM PTH with 100μM Diltiazem (DT) over a 46 hour time course (see S3 Video) (n = 3 observation fields±SD, with each field containing 30–40 cells, *p<0.05). Scale bar = 20μm.

RT-PCR was performed to determine the expression of the genes for these two calcium channels in differentiating IDG-SW3 cells over a time course of 1, 8, 15, 22, and 29 days. *Cacna1c* was elevated at day 8 and then decreased with time in culture, but PTH significantly increased expression at days 8, 15, 22, and 29 (Fig 6A). On the other hand, *Cacna1h* was increased with time in culture, peaking at day 22. PTH dramatically and significantly reduced expression at all time points (Fig 6B).

**Fig 7:**
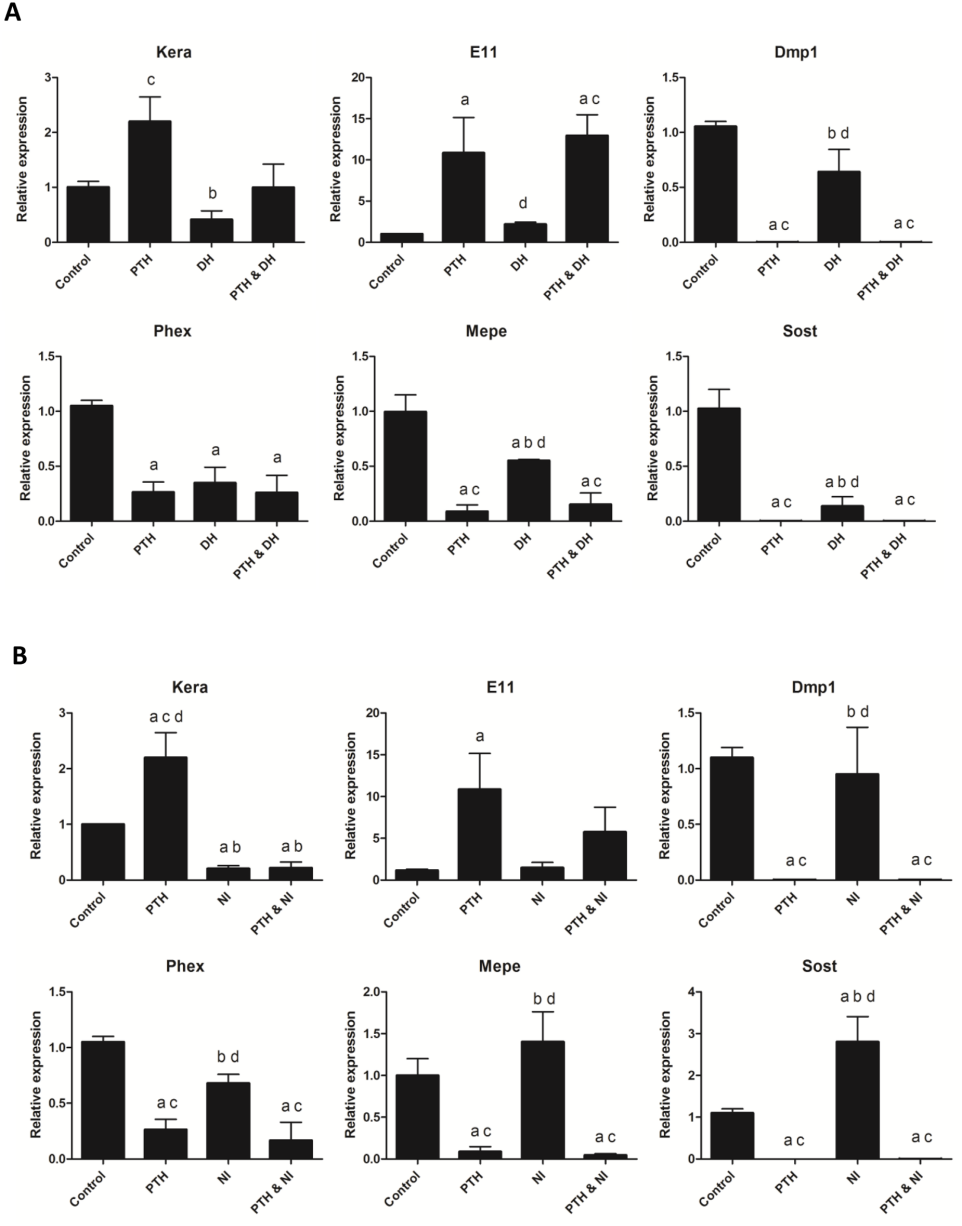
The calcium channel blockers Diltiazem and Nifedipine have no effect on PTH changes in gene expression except for Keratocan. (A) Real-time PCR analysis showing effects of Diltiazem on gene expression in mature IDG-SW3 cells. No effects on PTH changes in gene expression were observed (n = 3±SD, p<0.05 relative to control (a), PTH (b), DH (c) and PTH & DH (d)). (B) Effects of Nifedipine on gene expression. No effects of Nifedipine were observed on PTH induced gene expression except that Nifedipine inhibited PTH induction of *Kera*. (n = 3±SD, p<0.05 relative to control (a), PTH (b), NI (c) and PTH & NI (d)) Expression was normalized to *Actb*.

As shown by gene array analysis and RT-PCR, PTH upregulates the L-type channel over time in culture and down regulates the T-type channel during the same time period. This suggests that PTH could potentially utilize changes in calcium signaling to mediate its effects. We therefore examined whether L-type calcium channels may be involved in the PTH effects on cell morphology and motility through the use of two different L-type calcium channel inhibitors. Interference with L-type calcium channel activity in IDG-SW3 cells using the calcium channel blockers Nifedipine and Diltiazem was able to block the changes in cell morphology induced by PTH (Fig 6C). Blockage of L-type calcium channels with Diltiazem also attenuated the motility induced by PTH (Fig 6D and S3 Video). Interestingly, although antagonism of the L-type calcium channels inhibited the effects of PTH on cell morphology and motility, neither Diltiazem nor Nifedipine were able to completely block the downregulation of *Dmp1*-GFP expression in mature IDG-SW3 cells in response to PTH (Fig 6C). To determine whether this was true for the effects of PTH on other osteocyte markers, we examined the effects of Diltiazem or Nifedipine on PTH induced changes in expression of the osteocyte markers *Dmp1*, *Phex*, *Mepe* and *Sost* (Fig 7A and 7B). Similar to *Dmp1*-GFP expression, these markers were all still strongly downregulated by PTH in the presence of Diltiazem or Nifedipine. These L-type calcium channel inhibitors also had no effect on the ability of PTH to increase *E11* expression. Interestingly, the upregulation of *Kera* by PTH was blocked by Nifedipine. Together these data indicate that PTH regulation of the IDG SW3 cell shape change and motility is mediated via L-type calcium channel signaling, while its effects on osteocyte gene expression are mediated by a separate pathway.

## Discussion

As one might expect, the addition of PTH to IDG-SW3 cultures or primary osteocytes *ex vivo* had a significant effect on gene and protein expression. In our study, both primary osteocytes and IDG-SW3 cells responded to PTH by reverting away from a mature osteocyte phenotype. Such loss of differentiation of osteocytes in response to PTH was an interesting and unexpected finding. Sclerostin is expressed in day 28 SW3 cells very robustly at the RNA and protein level but was completely abolished by a single PTH treatment. Such a response to PTH has been observed previously in primary osteocytes [18,19] and provides further confirmation that IDG-SW3 cells mimic the behavior of mature osteocytes *in vivo*.

In addition to sclerostin, the downregulation of other osteocyte markers such as *Dmp1* and *Mepe* suggest that the PTH treated cells are losing their osteocyte phenotype. This was further confirmed by the upregulation of *Kera*, which has been shown to be expressed by osteoblasts but not osteocytes *in vivo* [43,44]. These results, in addition to the increased expression of the early osteocyte marker E11, may suggest that these cells are dedifferentiating to an earlier phenotype. However, RNA Seq analysis also showed that PTH induced downregulation of genes such as *Bglap* and *Col1a1*, which are indicative of active osteoblasts. Therefore, although these cells appear to be no longer behaving as mature osteocytes, they are not necessarily reverting back to an osteoblastic phenotype. This effect was not confined to *in vitro* cell lines as osteocyte enriched explants from mouse long bone also responded to PTH in a similar manner. PTH regulation of *Phex* has previously been observed in osteoblasts [45] and downregulation of *Dmp1* by PTH in osteocytes *in vivo* has been reported [18]. However, our results show the effect of a single treatment of PTH on the expression profiles of a range of early and late osteocyte marker genes at different stages of osteocyte differentiation. Furthermore, RNA Seq analysis of PTH-treated mature IDG-SW3 cell cultures showed a similar gene response profile to that observed in *ex vivo* cortical long bone osteocytes, showing the usefulness of these cells for studying the effects of osteoanabolic factors on osteocytes. Interestingly, a recent study by St John et al., [46] discovered that PTH negatively regulated the expression of many genes important for osteocyte differentiation in immature IDG-SW3 cells, while positively regulating genes which are normally downregulated during osteocyte differentiation. Therefore, PTH may act as an inhibitor of osteoblast to osteocyte transition, in addition to downregulating mature osteocyte marker gene expression.

One of the most intriguing findings from the present study was a dramatic alteration in the morphology of *Dmp1*-GFP positive IDG-SW3 cells, which was observed after PTH administration. Some of the cells changed from a typical dendritic osteocyte-like morphology to a highly elongated morphology. PTH has been shown to alter the osteocyte cytoskeleton and morphology *in vivo* [13,14] with the formation of lamellipodia like structures and an increase in microfilaments and microtubules. PTH also decreased the number of stress fibers in fetal rat calvarial osteoblasts *in vitro* via cAMP [47]. We also found that the PTH-induced morphological changes in IDG-SW3 cells were mimicked by the cAMP generator forskolin or the stable cAMP isoform 8-bromo-cAMP. The changes in morphology were associated with a dramatic increase in motility of the IDG-SW3 and primary cultures. PTH has been shown previously to promote cell motility in MG63 and Saos2 osteosarcoma cells [48,49). The related molecule, PTHrP has also been shown to stimulate migration in the MCF-7 breast cancer cell line [50] and giant cell bone tumor cells [51] but not in non-transformed bone cells. Interestingly, several motility associated genes were found to be upregulated by PTH treatment in IDG-SW3 cells, including epidermal growth factor receptor (*Egfr*). It was recently discovered that *Egfr* expression in mesenchymal cells was responsible for their migration in response to factors secreted by PTH stimulated osteoblasts and osteocytes [52]. Furthermore, the anabolic effects of PTH on bone formation *in vivo* were attenuated in a mouse model deficient in *Egfr* [52], indicating its importance in PTH induced bone formation. Another gene which was upregulated by PTH was *Ptn*, encoding pleiotrophin/heparin-binding growth-associated molecule (HB GAM). HB GAM has been demonstrated to induce motility of cells of the osteoblastic lineage and overexpression of HB GAM in a transgenic mouse model resulted in increased cortical and cortical bone volume [53]. This suggests that cell motility plays an important role in bone formation and that the anabolic effect of PTH on bone may be mediated by this increased motility.

In the IDG-SW3 cell culture model, some of the *Dmp1*-GFP positive cells are embedded within the mineralized matrix, but many of them are not completely surrounded by mineral and therefore may be representative of osteocytes that are partially embedded or embedded within the mineralized matrix but still close to the bone surface. The IDG-SW3 cells that were fully embedded did not appear to increase their motility in response to PTH. *In vivo*, it would therefore be expected that the PTH-induced increase in motility would be targeted towards partially embedded osteocytes and cells in the process of differentiating. Interestingly, it has been shown that osteocytes are able to remodel their perilacunar environment [54–57]. Furthermore, osteocytic osteolysis has been observed in response to continuous PTH treatment in rat cortical bone [58] and PTHrP treatment in mouse cortical bone [54]. Therefore it is conceivable that PTH treatment could potentially stimulate an embedded osteocyte to exit its lacuna and become motile if this was preceded by osteolytic osteolysis that was sufficient to release the cell from the constraints of its lacuna. While this might be possible for an osteocyte close to the bone surface, it remains unlikely that an osteocyte several cell layers down from the bone surface would be able to escape from its lacuna.

The increase in cell motility occurs concurrently with, and is likely linked to, the loss of the mature osteocyte phenotype in IDG-SW3 cells and primary osteocytes. Osteoblasts are known to be highly motile cells [35] and if these cells are reverting back to a more osteoblastic phenotype then it would be reasonable to assume that they would take on the properties of these cells. It is unknown what fate would befall the cells *in vivo* and whether they would be able to become fully functioning bone forming osteoblasts again, however, a recent study has shown that quiescent bone lining cells can be converted back to active osteoblasts by intermittent PTH treatment [59]. It has also been recently discovered that osteocytes are capable of de-differentiating into motile osteoblasts, when allowed to escape from their lacunae [60], suggesting a much greater plasticity of these terminally-differentiated cells than was previously imagined. This raises the possibility that PTH, via osteocytic osteolysis, facilitates the escape of embedding and recently embedded osteocytes. It is intriguing to speculate what functions these released cells might have, such as potentially providing an additional source of osteoblasts to increase bone formation, similar to the PTH-activated bone lining cells. However, it should be noted that the PTH-treated IDG-SW3 cells also had decreased expression of genes such as *Bglap* and *Col1a1*, suggesting that they are not behaving as mature, bone forming osteoblasts in the presence of PTH. Further studies would be required to determine whether such osteoblast markers are increased upon cessation of PTH treatment.

Cell motility can be regulated by many different intra and extracellular signaling pathways. Several pathways downstream of the PTH1R are known to be involved in cell morphology and motility, such as PKA [61,62], PKC [63,64], Epac [65,66] and Pi3K [67,68]. However, we found that none of these pathways were responsible for inducing morphological changes in the mature IDG-SW3 cells. The only inhibitors which could block these changes were those which targeted L-type calcium channels. Interestingly, we discovered that PTH treatment downregulated the expression of T-type and upregulated the expression of L-type calcium channel subunits in mature IDG-SW3 cells. It has previously been shown that L-type calcium channels are mainly expressed in osteoblasts whereas T-type channels are more highly expressed in osteocytes in comparison to osteoblasts [42]. This correlates with the de-differentiation of the mature IDG-SW3 cells in response to PTH. We also found that that L-type calcium channel blockers prevented the change in morphology induced by PTH and significantly reduced cell motility, suggesting the importance of the L-type calcium channels in mediating these effects. The loss of osteocyte phenotype in the IDG-SW3 cells was not blocked by the calcium channel blockers, however, suggesting that an alternative signaling pathway is responsible for these effects. Of course, these findings need to be confirmed in human cells.

In summary, we have shown that cells with the osteocyte phenotype respond to PTH treatment by losing their mature osteocyte phenotype. We have also shown that PTH changes the morphology and increases the motility of *Dmp1*-GFP positive cells in these cultures and in primary cells. This change in morphology and increased cell motility appears dependent on calcium signaling which is distinct from the effects of PTH on gene expression. As the mechanisms behind the anabolic effects of PTH are still being elucidated, these cells may play an important role in this process. We speculate that the capacity of PTH to enhance bone cell mobility may be one of the means whereby PTH accelerates bone turnover.

## Supporting Information

S1 Fig Generation of an *E11*/*gp38* flx plasmid construct. Two loxP sites were inserted into the *E11*/*gp38* exon 1 non-coding region and intron 1 respectively. A FRT-polII-neo^r^-FRT cassette was inserted before the 3’ loxP site for neomycin selection of ES cells. The final construct was confirmed by sequencing at every insertion site of the sub-cloning with five adjacent primers. As labeled in the map, the sequencing results of 5’ loxP, FRT, polII, 3’ FRT and loxP all matched theoretical expectation. The restriction mapping on the bottom left shows the correct fragmentation of the construct by *Sac*I: 0.5+6.4+6.6 kb, *Eco*RI: 0.5+9.8+3.2 kb, *Mfe*I: 13.5 kb, *Sal*I: 13.5 kb, *Spe*I: 7.6+5.9kb, and *Bam*HI: 0.8+1.4+4.5 +6.8 kb.
(DOCX)

S2 Fig Generation of mice with *E11*/*gp38* flx allele. After blastocyst injection and germline transmission, mice with the *E11*/*gp38* flx allele were confirmed by the *Eco*RI bands of a 296 bp product by PCR genotyping. The wild type allele showed a 190 bp product by PCR
(DOCX)

S1 Table GSEA analysis of genes downregulated by PTH in IDG-SW3 cells and primary *ex-vivo* osteocytes. (XLSX)

S2 Table GSEA analysis of genes upregulated by PTH in IDG-SW3 cells and primary *ex-vivo* osteocytes. (XLSX)

S1 Video PTH-induces an elongated shape and increased motility in mature IDG-SW3 cells. IDG-SW3 cells were differentiated for 28 days and the mineral was imaged by adding 0.5μg/ml alizarin red to the culture media as a vital stain for calcium. The left panel shows control cultures treated with the PBS vehicle and the right panel shows cultures treated with 50nM PTH 1–34. Time lapse images were captured using a widefield epifluorescence live imaging microscope every 30 minutes. Note the dramatic elongation of the *Dmp1*-GFP positive cells in the PTH treated culture and the increased motility of these elongated cells compared to the control.
(AVI)

S2 Video PTH-induces an elongated shape and increased motility in primary calvarial cells, which is not blocked by deletion of *E11/gp38*. Primary osteoblasts were isolated from *Dmp1*-GFP transgenic mice (left panel) and *Dmp1*-GFP transgenic mice on an *E11/gp38*-deficient background (right panel). The E11-deficient mice were homozygous for the *E11*fl/fl allele, which functions as a hypomorph, resulting in negligible expression of E11/gp38. The cells were differentiated for 21 days and then both cultures were treated with 50nM PTH 1–34. Time lapse images were captured using a widefield epifluorescence live imaging microscope every 30 minutes. Note that both the *Dmp1*-GFP control primary cells and the *Dmp1*-GFP/E11-deficient primary cells show similar cell elongation and increased motility in response to PTH, suggesting that the lack of E11/gp38 does not block the PTH induced response.
(AVI)

S3 Video Diltiazem blocks the elongated shape and increased motility in IDG-SW3 cells in response to PTH. IDG-SW3 cells were differentiated for 21 days and then treated with 50nM PTH 1–34 (left panel) or PTH + 100μM Diltiazem Hydrochloride (right panel). Time lapse images were captured using a widefield epifluorescence live imaging microscope every 30 minutes. Note the reproducible elongation of *Dmp1*-GFP positive cells and increase in motility with PTH treatment, which is blocked in the presence of diltiazem.
(AVI)
