# Supplementary material for: Parathyroid Hormone Induces Bone Cell Motility and Loss of Mature Osteocyte Phenotype through L-Calcium Channel Dependent and Independent Mechanisms
Source: PLoS One. 2015 May 5;10(5):e0125731. doi: 10.1371/journal.pone.0125731 (PMC4420268; doi:10.1371/journal.pone.0125731)
Supplement: S1 Fig — Two loxP sites were inserted into the E11/gp38 exon 1 non-coding region and intron 1 respectively. A FRT-polII-neor-FRT cassette was inserted before the 3’ loxP site for neomycin selection of ES cells. The final construct was confirmed by sequencing at every insertion site of the sub-cloning with five adjacent primers. As labeled in the map, the sequencing results of 5’ loxP, FRT, polII, 3’ FRT and loxP all matched theoretical expectation. The restriction mapping on the bottom left shows the correct fragmentation of the construct by SacI: 0.5+6.4+6.6 kb, EcoRI: 0.5+9.8+3.2 kb, MfeI: 13.5 kb, SalI: 13.5 kb, SpeI: 7.6+5.9kb, and BamHI: 0.8+1.4+4.5 +6.8 kb. (DOCX) [file pone.0125731.s001.docx]

**S1_Fig.**
