# Supplementary figures and images for: Parathyroid Hormone Induces Bone Cell Motility and Loss of Mature Osteocyte Phenotype through L-Calcium Channel Dependent and Independent Mechanisms
Source: PLoS One. 2015 May 5;10(5):e0125731. doi: 10.1371/journal.pone.0125731 (PMC4420268; doi:10.1371/journal.pone.0125731)

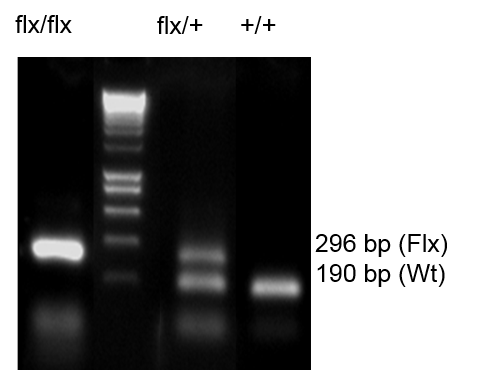


S2_Fig.

Supplement: S2 Fig — After blastocyst injection and germline transmission, mice with the E11/gp38 flx allele were confirmed by the EcoRI bands of a 296 bp product by PCR genotyping. The wild type allele showed a 190 bp product by PCR (DOCX) [file pone.0125731.s002.docx]
